# Comparison of Foraging Strategies and Effects of the Wapiti and Siberian Roe Deer on Japanese Yew

**DOI:** 10.1002/ece3.70451

**Published:** 2024-10-23

**Authors:** Xianzhe Wang, Jianan Feng, Yang Hong, Hairong Du, Minghai Zhang

**Affiliations:** ^1^ College of Wildlife and Protected Area Northeast Forestry University Harbin China

**Keywords:** *Capreolus pygargus*, *Cervus elaphus*, foraging strategy, kernel density estimation, maximum entropy model, *Taxus cuspidata*

## Abstract

The foraging strategies of sympatric ungulates with similar ecological niches are important for understanding ecological niche differentiation, resource utilization, competition, and coexistence and for understanding the ecological impacts on plant communities in the ecosystem. The behavior of the wapiti (*Cervus elaphus*) and Siberian roe deer (*Capreolus pygargus*) foraging on Japanese yew (*Taxus cuspidata*) has affected its succession and renewal in the northeastern forests of China, which has become an urgent problem for the relevant departments. This study analyzed the foraging strategies of the wapiti and Siberian roe deer on Japanese yew from July 2021 to January 2024 using field investigations and infrared camera monitoring in the Muling National Nature Reserve, Heilongjiang Province, China. It was found that the wapiti and Siberian roe deer have different foraging strategies in terms of time, space, and behavior. Temporally, they both preferred to forage for the saplings of the Japanese yew during the winter season, the degree of overlap in foraging rhythms was medium (Dhat1 = 0.67), and the diurnal foraging activity index (*D*_RAI_) of the wapiti was larger than that of the Siberian roe deer. Spatially, the suitable foraging habitat of the Siberian roe deer was twice that of the wapiti, and their overlap was low in the location and direction of saplings and the distance of the seed tree. Behaviorally, the foraging intensity of the wapiti was high, and that of Siberian roe deer was low. Foraging reduced the average primary branch height, number of new branches, and length of lateral branches of saplings, and the influence of the wapiti was significantly greater than that of the Siberian roe deer. This study provides a scientific basis for solving the conservation and management problems of the deer animals foraging on Japanese yew and contributes to further understanding of the competition‐coexistence mechanism of sympatric species.

## Graphical abstract

The behavior of wapiti (*Cervus elaphus*) and Siberian roe deer (*Capreolus pygargus*) foraging Japanese yew (*Taxus cuspidata*) that has affected its succession and renewal in China. We found that wapiti and Siberian roe deer have different foraging strategies in time, space, and behavior. This study provides a scientific basis for solving the conservation and management problems of wapiti and other deer species foraging on Japanese yew and contributes to further understanding the competition‐coexistence mechanism of sympatric species.

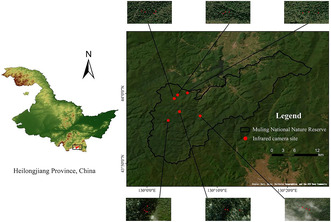

## 1 Introduction

The foraging strategy of ungulates is a key link between primary production and food webs (Elsayed and Din 2019). It influences the structure and trends of changes in animal communities (Schoener 1974; Wang, Wan, and Zhong 2001), species distribution, resource utilization, and population dynamics (Heleno, Devoto, and Pocock 2012; Tylianakis et al. 2010; Zanni et al. 2021) and plays a major role in maintaining structural integrity and balance (Ge et al. 2012; Kasiringua, Proche, and Kopij 2020; Wang, Yang, et al. 2019).

There are certain interactions between plants and animals (Li and Liu 2002). Studies have found that the foraging of ungulates affects the growth of plants, seriously leads to the failure of renewal, and has a negative effect on interspecific competition (Bödeker et al. 2021; Konôpka et al. 2021; Kamler et al. 2010; Vacek 2017), for example, spruce (*Picea abies*) seedlings grow slowly when foraged by roe deer (*Capreolus capreolus*) (Bergquist, Bergström, and Zakharenka 2003). Plants also develop defense mechanisms to protect themselves, for example, sheep (*Ovis aries*) reduce their intake when the flavonoid content of their food is too high (Sun 2008).

Interactions between sympatrically distributed animals and plants have been a classic proposition in evolutionary ecology, and the ecological impacts produced by animals on plants have been a popular topic of discussion in conservation biology and biodiversity conservation (Feng 2022). In northeastern China, Japanese yew (*Taxus cuspidata*) is a tertiary relict plant (Liu 2007), which is a wild and endangered class I nationally protected plant. The wapiti (*Cervus elaphus*) and Siberian roe deer (*Capreolus pygargus*) are both protected animals and are the main prey of the Amur tiger (*Panthera tigris*) and Amur leopard (*Panthera pardus orientalis*). The wapiti, Siberian roe deer, and Japanese yew are, therefore, all the main protection species that need to be focused on for the protection and management of national parks and nature reserves in northeastern China.

Previous studies have found that both the wapiti and Siberian roe deer foraged on Japanese yew under environmental stresses such as low temperatures, snow cover, and lack of food resources in winter (Feng 2022). The Japanese yew accounts for 13% of the winter food composition of the wapiti, which leads to significant damage to the saplings (Zhu et al. 2019), resulting in a dramatic decline in the number and range of Japanese yew (Diao et al. 2020; Yang 2018). As the number of saplings decreased, ungulates such as wapiti chose other plants as food, and the Japanese yew population gradually recovered. On the other hand, the number of ungulates, such as the wapiti, is also decreasing in reserves due to human activities such as long‐term forest logging, illegal poaching, and road construction in the past (Dixon et al. 2021; Sarula, Zhang, and Gao 2019). The decrease in the number of natural enemies has increased the number of Japanese yew. Protecting wildlife like the wapiti, without affecting the succession and renewal of the Japanese yew forests, has become an urgent problem for the management department of nature reserves.

Therefore, this study used the Kernel Density Estimation, Maximum Entropy Model, infrared camera monitoring technology, and field investigation method to compare the foraging strategies of two ungulates on Japanese yew from three aspects of time, space, and behavior characteristics and highlighted their different impacts on Japanese yew in more detail. It is helpful to understand the niche differentiation model, spatial distribution and resource utilization characteristics, competition, and coexistence mechanism of the two species. The results not only contribute to further understanding of the competition‐coexistence mechanism of sympatric species but also enrich the study of animal and plant interaction and co‐evolution.

## 2 Materials and Methods

### 2.1 Study Area

The study area is located in the Muling National Nature Reserve, in the southern part of the Laoyeling, in the northern part of the Changbai Mountain (130°00′–130°28′ E, 43°49′–44°06′ N). The south is bordered by the Tianqiaoling Forestry Bureau of Jilin Province, the east is bordered by the Suiyang Forestry Bureau of Heilongjiang Province, and the west, north, and northeast are connected with the Gonghe Management Office, Shuangning Forest Farm, and Yangmuqiao Forest Farm of Muling Forestry Bureau, with a total area of 356.48 km^2^ (Feng 2022). The mountains in this area are low, with altitudes ranging from 500 to 900 m, and the landforms are distributed in bands. The climate is the typical temperate continental climate with an average annual temperature of approximately −2°C. The rainy season is short, and precipitation is concentrated, with an average annual rainfall of 514 mm and an annual frost‐free period of 130 days (Tian et al. 2022).

The forests in the reserve are primarily coniferous, broad‐leaved forests, mixed coniferous and broad‐leaved forests, and thickets, with 839 plant species belonging to 113 families and 373 genera. The dominant species are red pine (*Pinus koraiensis*), purple linden (*Tilia amurensis*), and red‐barked spruce (*Picea koraiensis*). There are 42 vertebrate species belonging to 6 orders and 14 families, represented by the Amur tiger, Amur leopard, wapiti, sika deer (*Cervus nippon*), Asian black bear (*Ursus thibetanus*), and wild boar (*Sus scrofa*) (Feng 2022).

### 2.2 Methods

#### 2.2.1 Field Investigation

Areas with a relatively concentrated distribution of Japanese yew were selected for field investigations every winter from 2021 to 2024. The saplings were divided into foraging and control groups.

When recording the foraging situation of each saplings, according to (1) infrared camera photos and videos; (2) the size and characteristics of snow footprints; (3) the height and characteristics of foraging traces distinguish wapiti from Siberian roe deer foraged saplings. Those who had been fed by deer were defined as the foraging group, and those who had not been fed were defined as the control group.

During the survey, the saplings that were foraged on were labeled and their height, length of lateral branches, number of branches foraged, total number of branches, and information about the surrounding environment were recorded (Feng 2022). Saplings that have not been eaten by cervids were selected as controls in the corresponding areas, and the plant height and number of branches were recorded. The height of the primary branch growth, the number of new branches, and the length of lateral branch growth for each sapling were recorded every summer from 2021 to 2024. A total of 1947 foraging traces were observed. Among them, 1235 were of the wapiti and 712 of the Siberian roe deer.

#### 2.2.2 Infrared Camera Monitoring

**FIGURE 1:**
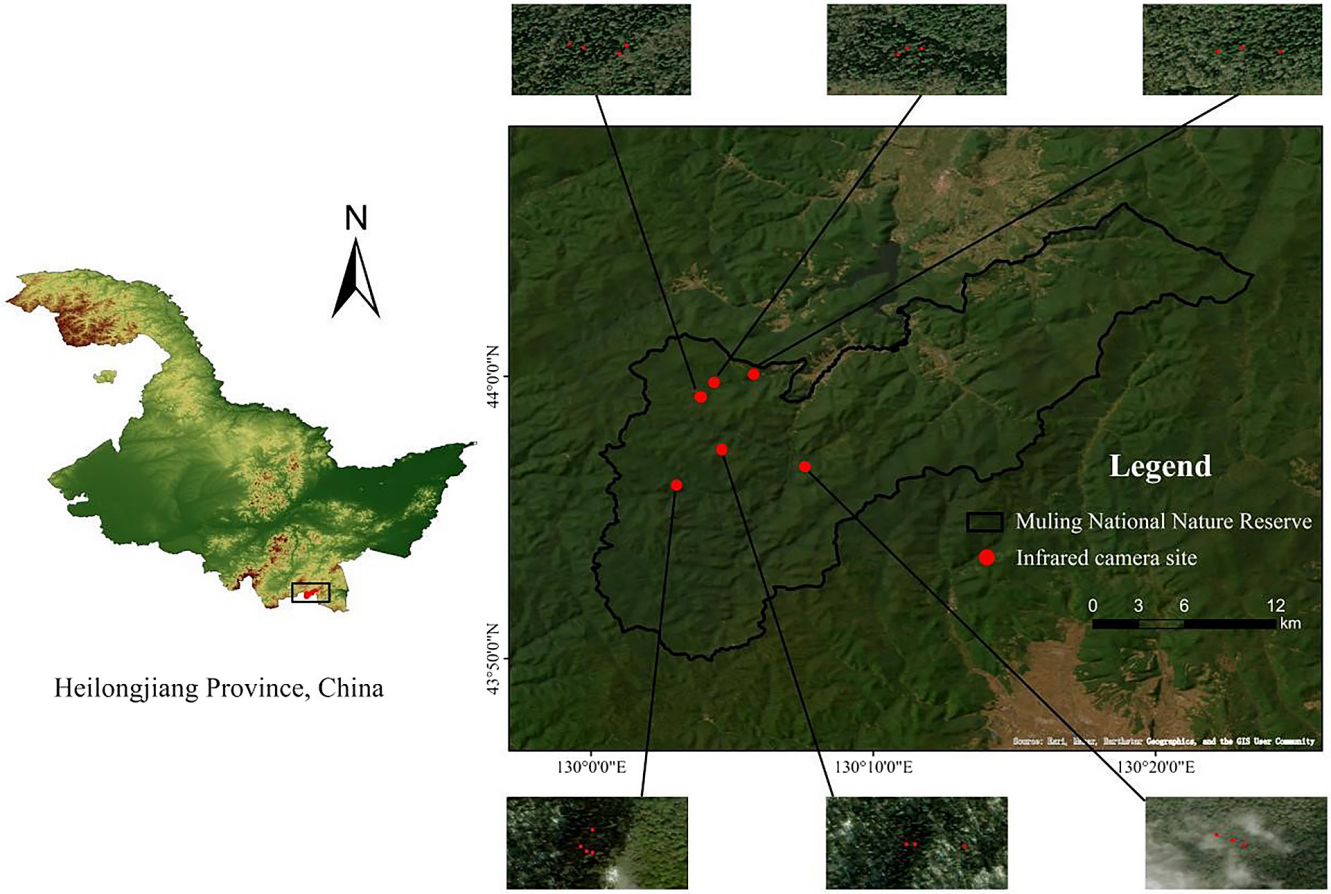
Location of the Muling National Nature Reserve in northeast China.

According to the results of previous field surveys in conservation (Feng 2022), a total of 20 infrared cameras were set up in 8, 10, 13, 30, 41, 45, and 55 forest classes (Figure 1), and the site selection of infrared cameras should not only meet the distribution of Japanese yew seedlings, but also meet the frequent activities of wapiti and Siberian roe deer. The height of the camera from the ground is 30–90 cm, and the selection of the height of the camera can not only photograph the entire Japanese yew saplings, but also ensure that the height is in line with the feeding height of wapiti and Siberian roe deer. The height of the infrared cameras is adjusted to ensure that the entire sapling is photographed. Camera parameter settings were as follows: photo (three photos) + video (15 s). After the camera was installed, the weeds in front of the shooting area were removed to prevent misshooting and blocking the lens (Zhao et al. 2020). The monitoring period lasted 2.5 years.

#### 2.2.3 Environment Variable Data

Divide environment variables into four categories:

1. Land‐use types: Scrub, evergreen coniferous forest, deciduous coniferous forest, mixed coniferous forest, deciduous broad‐leaved forest, and farmland. The vegetation type variables were extracted from the vector forest phase map of the protected area, and a Euclidean distance layer was generated for each raster to the nearest vegetation type.
2. Anthropogenic disturbance factors: Rural roads and forest trails were obtained by vectorization in ArcGIS 10.8 software using forest topographic maps. According to the vector layer of settlement and road distribution in the protected area, buffer zones of 500 and 200 m were established, respectively, and the Euclidean distance layer from the center point of each raster to the nearest buffer of the two was calculated as artificial interference variables.
3. Topographic factors: From the resource and environmental data cloud platform of the Resource and Environmental Science Data Center of the Chinese Academy of Sciences (http://www.resdc.cn) Download the 30 m × 30 m study area Digital Terrain Elevation Model (DEM) data to make the elevation, slope, and aspect of the study area were extracted using the Spatial Analysis Module of ArcGis 10.8.
4. Water sources: Streams were obtained by vectorization in the ArcGIS 10.8 software using forested topographic maps. According to the river distribution vector layer of the protected area, the Euclidean distance layer from the center point of each raster to the nearest water source is generated.

#### 2.2.4 Data Analysis

##### 2.2.4.1 Foraging Rhythms of the Wapiti and Siberian Roe Deer

The kernel density estimation method assumes that animal behavior is distributed in continuous time with a period of 24 h, and its behavior events are randomly sampled in the continuous time distribution. This method does not attach any assumptions to the data distribution and is a method to study the data distribution characteristics from the data sample itself (Ridout and Linkie 2009). It can calculate the main parameters of daily activity rhythm such as mean activity duration, median, standard deviation, variance, and density of animals. When an individual appears in front of the camera once, no matter how many consecutive shots are taken, it is recorded as ‘one independent capture event’. When the same individual is identified and captured repeatedly, the time interval less than 30 min is recorded as one independent capture event, and the time interval greater than 30 min is recorded as two independent capture events (Ma et al. 2020). According to the statistics of each individual effective feeding times of wapiti and roe deer in Siberia, the daily feeding rhythm, monthly feeding rhythm, and cold and warm season feeding rhythm of these two deer were calculated.

Taking the average time of sunrise and sunset of the local year as the demarcation, the day was divided into day and night, with reference to the local climate. May to October was classified as the warm season, and November to April was classified as the cool season. The daily and annual foraging rhythms were analyzed in this study by using the kernel density estimation method (Chen, Shu, and Xiao 2019) with the overlap package and activity package of R. Delta4 values were selected for calculations when both the sample sizes compared were ≥ 75, and the Delta1 values were selected when the size was < 75 (Jiang et al. 2023). In addition, the degree of temporal overlap between the activities of the two species was classified as high overlap when Delta > 0.75, medium overlap when Delta < 0.50 < Delta < 0.75, and low overlap when Delta < 0.50 (Monterroso, Alves, and Ferreras 2014).

Relative Activity Intensity Index (RAI) is (frequency of animal activity photographed in a given period/total number of animal activity photographed) × 100% (Li 2022). The foraging patterns of the wapiti and Siberian roe deer on Japanese yew saplings were analyzed by calculating the RAI for the day and night (*D*_RAI_), month (*M*_RAI_), and season (*S*_RAI_) (Liu, Zhao, and Wang 2022). The formulas were as follows: *D*_RAI_ = (*D*_*ij*_/*N*_*i*_)100, *M*_RAI_ = (*M*_*ij*_/*N*_*i*_)100, and *S*_RAI_ = (*S*_*ij*_/*N*_*i*_)100. *D*_*ij*_ denotes the number of independent foraging occurrences of species *i* in the time period *j* (both day and night), *M*_*ij*_ denotes species *i* in the time period *j* (monthly), and *S*_*ij*_ denotes species *i* in the time period *j* (cold and warm seasons). *N*_*i*_ represents the sum of independent foraging times of species *i* during the shooting period.

##### 2.2.4.2 Foraging Habitats Suitability Prediction

We collected and collated the data of field survey and infrared camera monitoring in the reserve and obtained 69 feeding points of wapiti and 82 feeding points of Siberian roe deer. Sampling bias correction based on the occurrence point: To avoid sampling bias resulting in overfitting of the model, Spatially Rarefy Occurrence Data for SDM tool in SDM Tools of ArcGIS software was used to filter all feeding points of wapiti and Siberian roe deer (Brown 2014), so that the distance between any two points was not less than 500 m. Each grid randomly retains one of its distribution points and eliminates the rest. In the end, 17 wapiti feeding sites and 23 Siberian roe deer feeding sites were retained and used. The selected sites and all environmental variables in Section 2.2.3 were used to predict suitable foraging habitats; 75% of the points were used for the construction of the model, 25% were used for validation, and the model was run for 10 cycles. This was evaluated using the cutting method and its comprehensive contribution. The difference of the area under the curve (AUC) in the receiver operating characteristic curve (ROC) analysis between the modeling data and the test data was used to evaluate the real prediction ability of the model (Qin et al. 2015). It is generally believed that an AUC value < 0.5 indicates that the prediction results are not referable; 0.5–0.7 indicates that the prediction ability is average, 0.7–0.9 indicates that the prediction ability is good, and 0.9–1 indicates that the prediction ability is excellent (Huang, Zhao, and Shi 2020). All operations were performed using MaxEnt 3.3.

The suitable habitats for the Japanese yew and ungulates were reclassified using ArcGIS 10.8. The average value of the maximum training sensitivity and specificity after 10 modes of operation was used as the threshold for the distribution of suitable foraging habitats (Li 2022). The habitats of Japanese yew were divided into two levels—0–0.153 was unsuitable habitats, and 0.153–1 was suitable. The foraging habitats of the ungulates were divided into three levels—0–0.55 was unsuitable foraging areas, 0.55–0.75 was generally suitable, and 0.75–1 was suitable.

##### 2.2.4.3 Foraging Characteristics of the Two Ungulates and Their Effects on Saplings

Based on the results of Feng (Feng 2022), we classified the degree of foraging into mild (0%–35%), moderate (36%–70%), and severe (71%–100%). The calculation method is as follows: the number of branches eaten by a single seedling/the total number of branches in a single seedling × 100%.

Taking Japanese yew as the center, the study area was extended in eight directions—east, west, south, north, northeast, southeast, northwest, and southwest. The sapling points were recorded along with the information on whether foraging occurred or not within 50 m in each direction. A total of 71 points were collected, and in order to explore the characteristics of the distance between the saplings and mother trees of Japanese yew foraged by cervids, they were plotted using the fmsb package in R.

The characteristics of ungulates foraging on saplings were summarized using infrared camera data. The *t*‐test and Kolmogorov–Smirnov test were used to analyze the following: (1) whether the monthly relative abundance index of the two ungulates foraging for young trees was different; (2) whether there was a difference in the distance between the two ungulates foraging on the young tree and the mother tree; and (3) whether the growth height of the main branches, the number of new branches, and the growth amount of lateral branches after foraging by the two ungulates were different. All operations were performed using R 4.2.1.

## 3 Results

### 3.1 Foraging Time Strategies of the Two Ungulates

**FIGURE 2:**
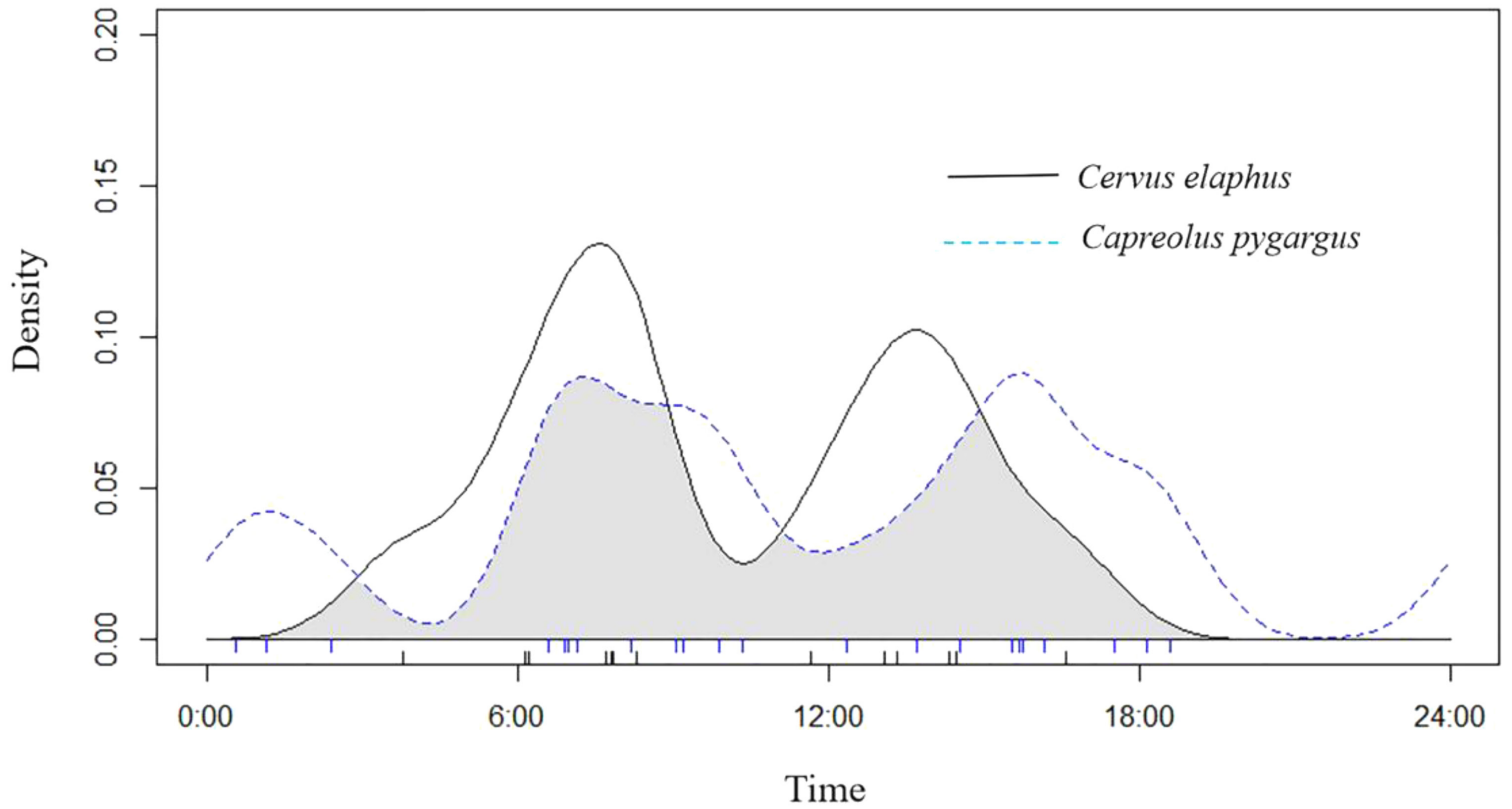
Diurnal foraging rhythm of two species of deer Japanese yew saplings.

The diurnal foraging activity index of the wapiti was higher than that of the Siberian roe deer (*D*_RAI_ = 92.3%), and the nocturnal feeding activity index of the Siberian roe deer was also higher. There were two peaks in the diurnal activity rhythms of the wapiti foraging on saplings, which were from 7:00 to 8:00 and from 14:00 to 15:30, with the overall shape of an “M,” and the size of the peaks varied. There were three peaks for the roe deer, from 6:30 to 9:30, 15:30 to 18:30, and 24:00 to 2:00, with the entire peak being wavy. In terms of the degree of overlap, the highest overlap indices were observed at 7:00 and 15:00, and temporally, the degree was moderate (Dhat1 = 0.67) (Figure 2).

**FIGURE 3:**
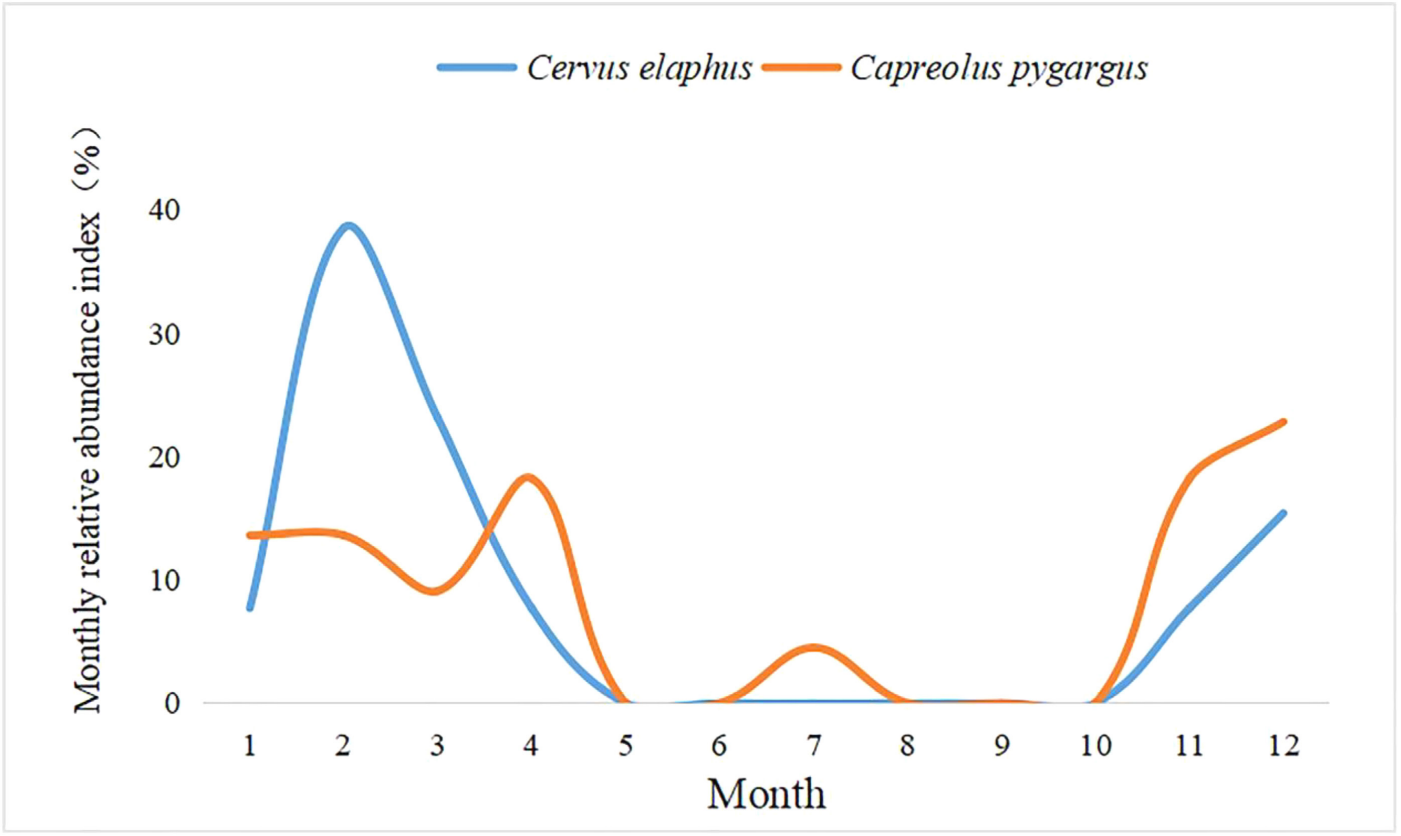
Monthly relative abundance index of two species of deer foraging on Japanese yew saplings.

The monthly relative abundance index (*M*_RAI_) of wapiti foraging demonstrated an unimodal trend of first increasing and then decreasing, reaching its peak in February (38.5%), which was approximately twice that of roe deer (22.8%), whereas there was no obvious pattern for the latter as a whole (Figure 3). This indicated that the two ungulates foraged on Japanese yew saplings seasonally. The results demonstrated that there was a difference in the relative multiplicity index (*S*_RAI_) of foraging between them in the cold season (*p* < 0.05).

### 3.2 Spatial Strategies of Foraging in Two Deer Species

#### 3.2.1 Habitat Suitability of the Japanese Yew

**FIGURE 4:**
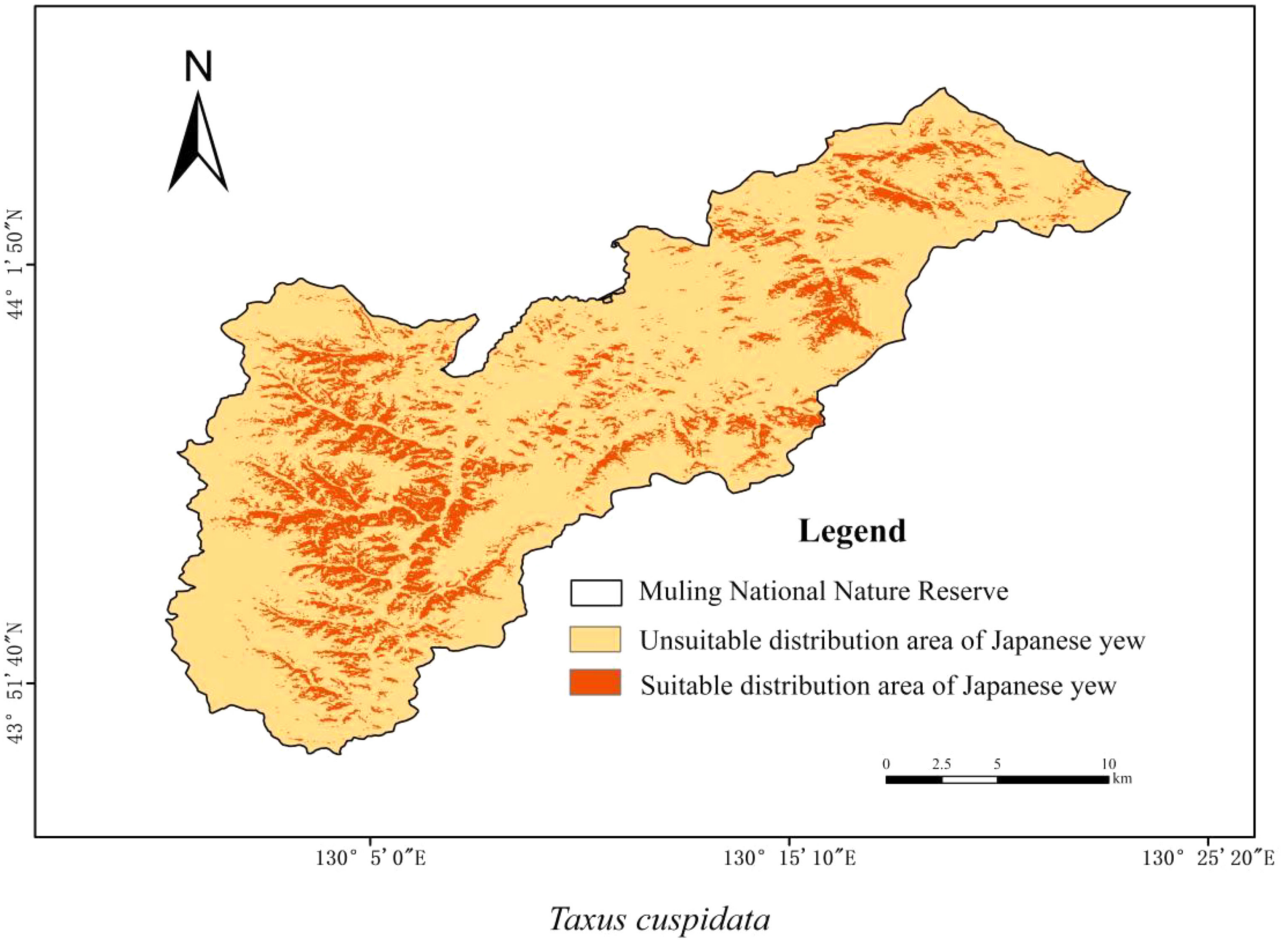
Distribution map of habitat suitability for Japanese yew.

The result of the ROC demonstrated that the AUC was 0.92 ± 0.04, and the prediction reached the “good” standard. The habitat suitability analysis showed that the area unsuitable for the study was 292.71 km^2^, which is 82.11% of the total area, and the suitable area was 63.77 km^2^ accounting for 17.89% of the total area (Figure 4).

#### 3.2.2 Suitable Foraging Habitats of the Two Ungulates

The results of ROC showed that the AUC was ≥ 0.8, reaching the “good” standard (Table 1). This indicates that the MaxEnt model was able to predict the potential foraging distribution area.

**TABLE 1:** MaxEnt results of red deer and Siberian deer foraging on northeast Taxus habitat.

Species	Feeding points	Spatial post‐screening loci	AUC	Standard deviation
Wapiti	69	17	0.894	0.035
Siberian roe deer	82	23	0.907	0.026

The model results demonstrated that vegetation type was the most beneficial environmental factor in predicting suitable foraging habitats for the two ungulates, and the contribution rate of the wapiti was 50%, whereas that of the Siberian roe deer was 40.4%. Among these, evergreen broadleaf and mixed conifer forests had the greatest impact, whereas deciduous broadleaf forests had the least. Aspect (21.9%) and altitude (13.9%) were the key factors affecting the foraging habitat of the wapiti, followed by slope (5.8%), distance from road (4.8%), and distance from water (3.6%). Aspect (23%) and slope (21.5%) were also key factors for Siberian roe deer; altitude (9.4%), distance from roads (3.3%), and distance from water sources (2.4%) contributed the least (Table 2).

**TABLE 2:** The importance of environmental variables.

Environmental variables	Wapiti	Siberian roe deer
Vegetation types	0.500	0.404
Aspect	0.219	0.230
Slope	0.058	0.215
Altitude	0.139	0.094
Distance from road	0.048	0.033
Distance to water source	0.036	0.024

**FIGURE 5:**
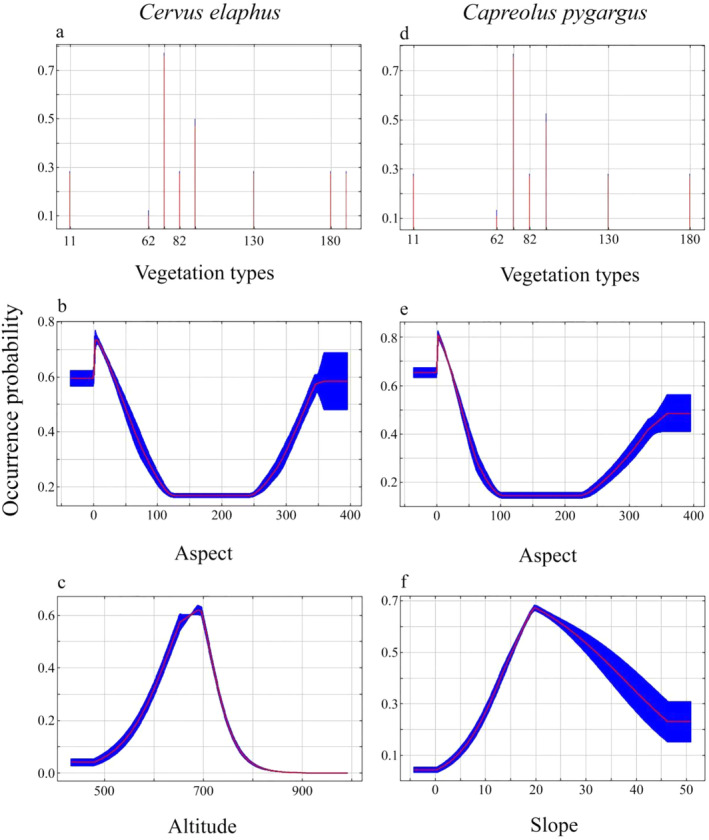
Response curves of major environmental variables. Among the vegetation types, 11 are covered by herbaceous plants; 62 is a deciduous broad‐leaved forest; 72 is an evergreen coniferous forest; 82 is a deciduous coniferous forest; 92 is a mixed coniferous and broad‐leaved forest; 130 is grassland; 180 is a wetland.

The prediction results demonstrated that the wapiti foraged on saplings at an altitude of 700 m, with slopes of 20°–30°, aspects to the north, and far away from the road (2000 m) and the water source (3500 m) in the reserve. The foraging habitat suitability gradually increased with increasing distance from the evergreen coniferous forest and mixed coniferous forest. However, the Siberian roe deer preferred an altitude of 650 m, with slopes of 15°–20°, and the other factors were similar to the wapiti (Figure 5).

**FIGURE 6:**
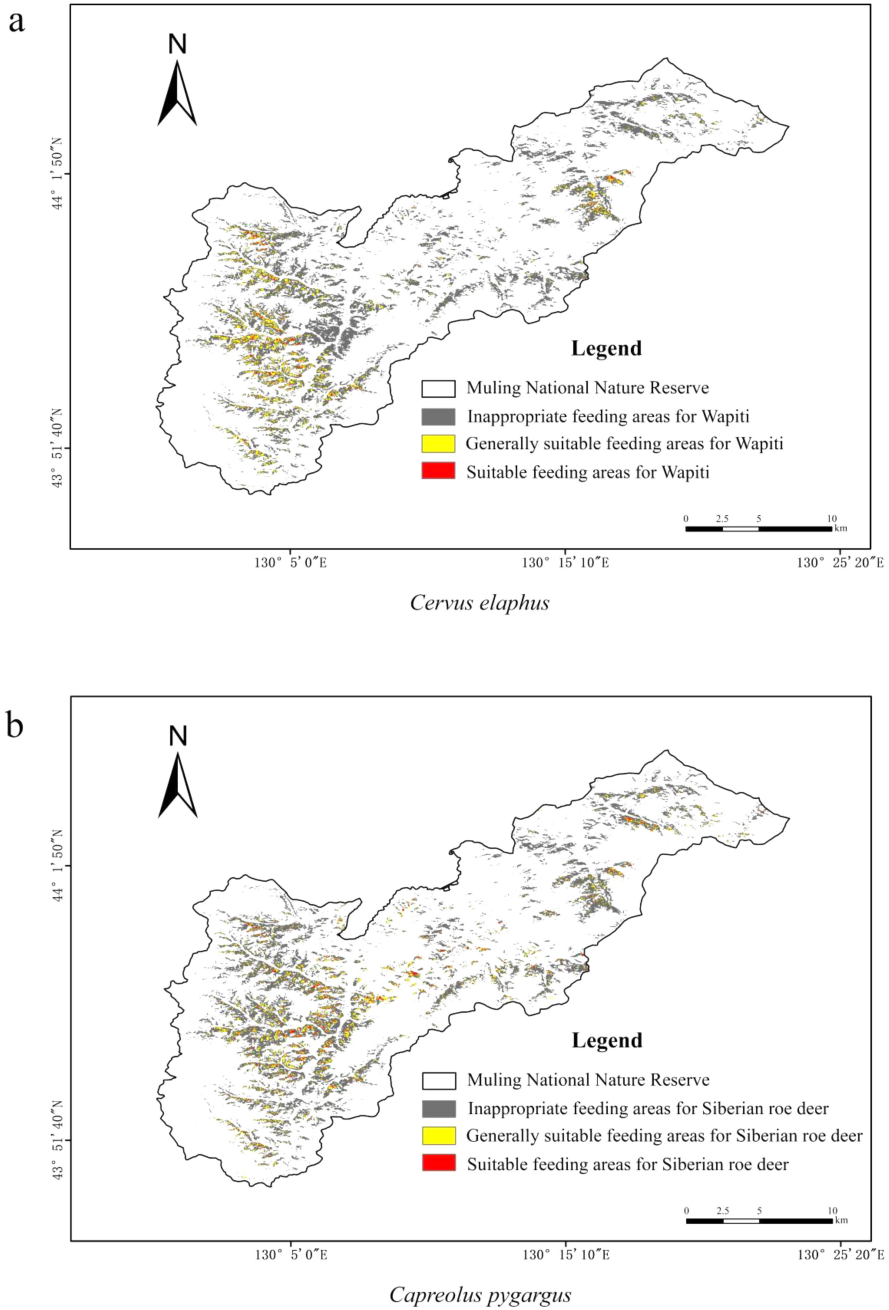
Suitable distribution map for foraging on saplings of Japanese yew.

The suitability analysis of foraging habitat demonstrated that the area of suitable, average, and unsuitable habitats for the wapiti foraging in the study area was 0.98, 5.6, and 57.1 km^2^, accounting for 1.53%, 8.78%, and 89.69% of the total area of the study area, respectively. The areas of suitable, average, and unsuitable habitats for Siberian roe deer foraging were 1.69, 6.5, and 55.67 km^2^ in that order, accounting for 2.65%, 10.05%, and 87.30% of the total area of the study area, respectively (Figure 6) (Table 3). According to the analysis of habitat overlap between wapiti and Siberian roe deer, about 65% of the habitats suitable for eating Japanese yew in winter are also suitable for eating areas of Siberian roe deer, which is mainly reflected in the utilization of the mixed forests in the protected area.

**TABLE 3:** Habitat suitability analysis of two species of deer foraging on Japanese yew.

Species	Unsuitable area	General application area	Suitable area
Wapiti	57.1 km^2^	5.6 km^2^	0.98 km^2^
Siberian roe deer	55.67 km^2^	6.5 km^2^	1.69 km^2^

### 3.3 Characteristics of Foraging Behavior of the Two Ungulates

#### 3.3.1 Foraging Distance

**FIGURE 7:**
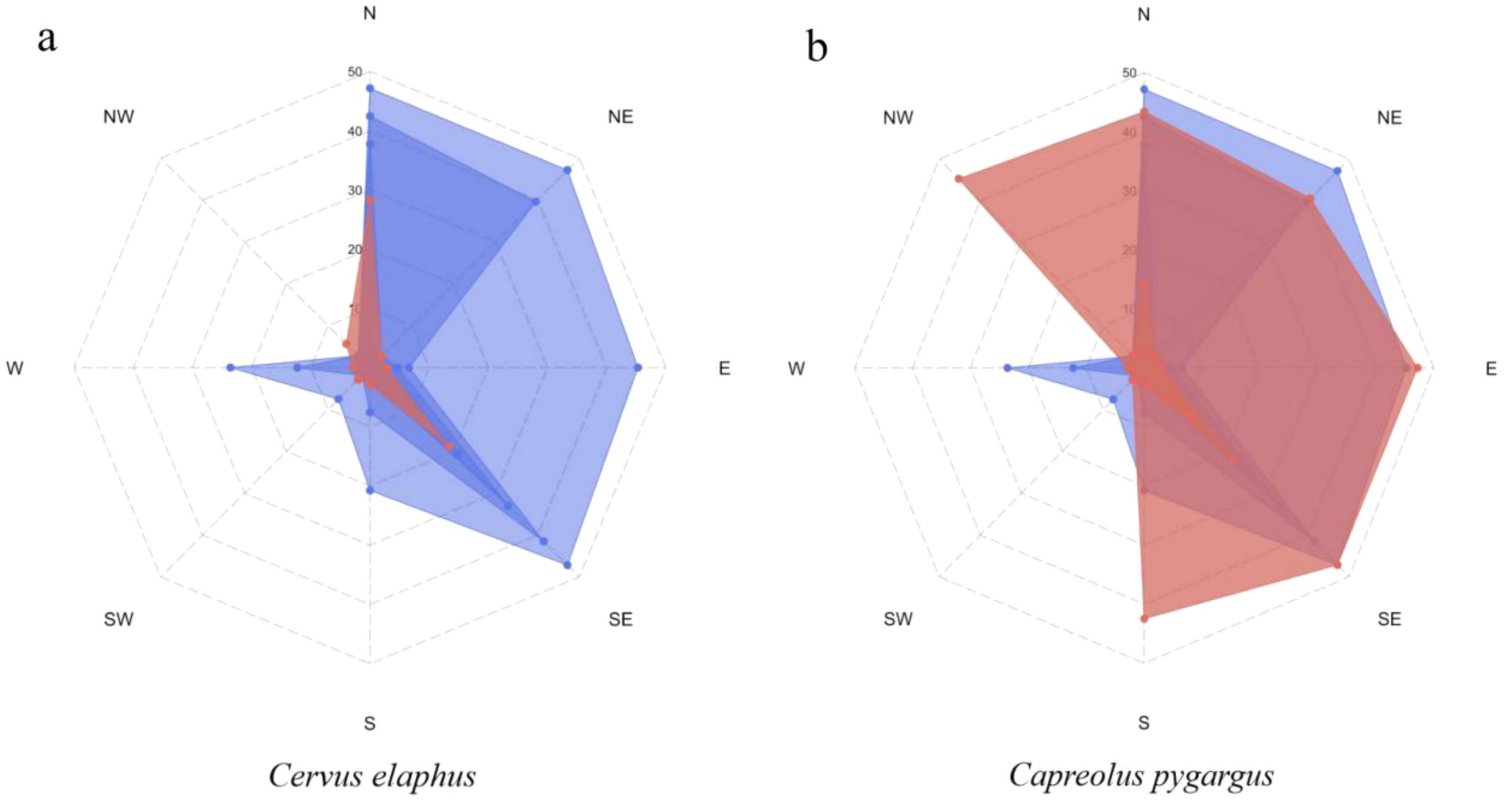
Relationship between the distance between foraging Japanese yew saplings and mother trees. The red is the foraging area, and the blue is the area where Japanese yew grows.

The wapiti preferred to forage on saplings located close to mature trees, ranging from 1.5 to 30 m in the northwest and 295° to southeast 140°. The Siberian roe deer presented some variability (*p* < 0.05); their feeding distance was longer, ranging from 8 to 50 m and the range was wider, from 315° to south 180° (Figure 7).

#### 3.3.2 Foraging Intensity

**FIGURE 8:**
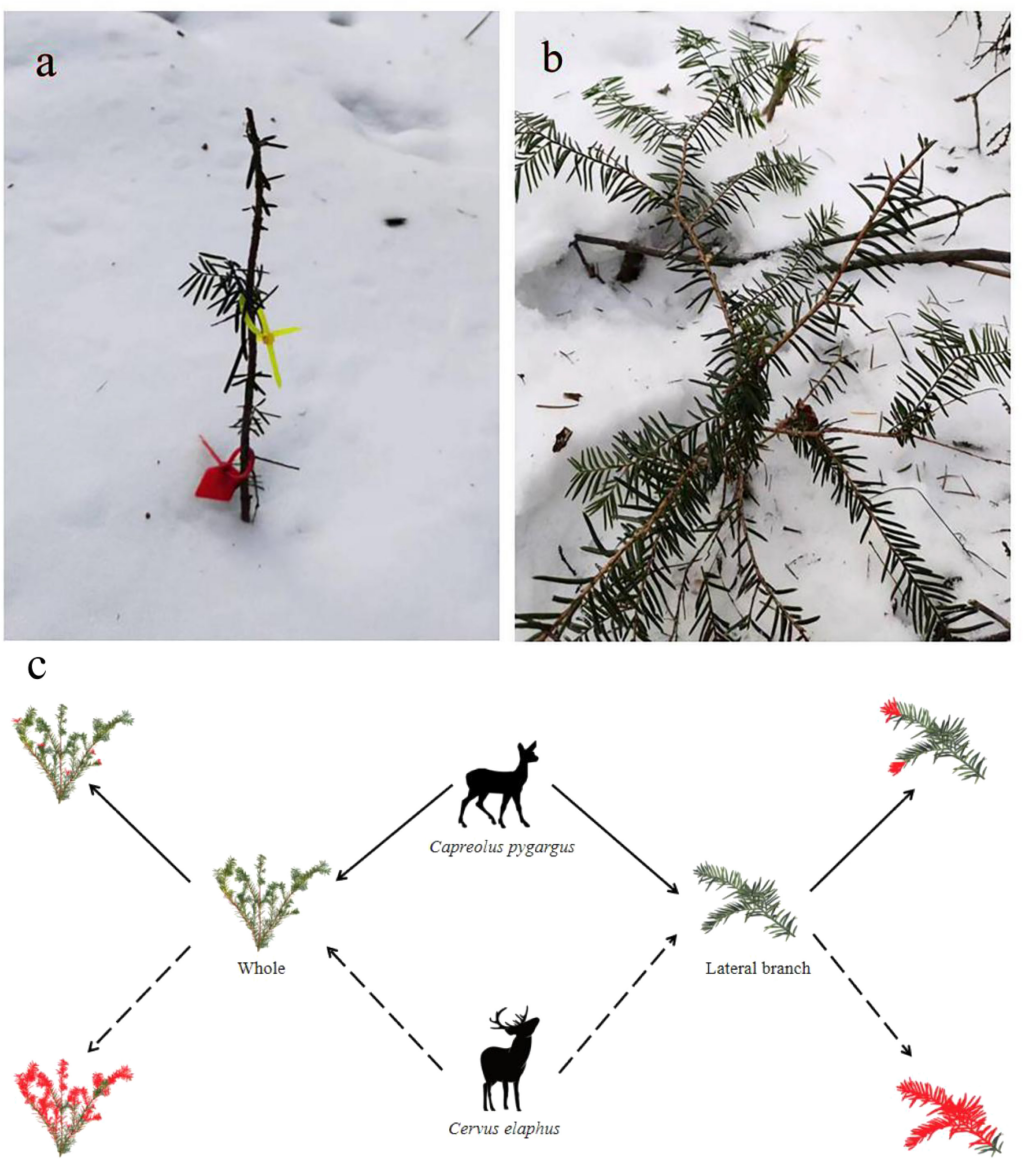
The degree to which two species of deer animals forage on Japanese yew saplings. (a) Japanese yew after being foraged by wapiti; (b) Japanese yew after being foraged by siberian roe deer; (c) a simulated image of an intact Japanese yew sapling and one of its lateral branches after being foraged by two species of deer. Red represents the foraging part.

According to the infrared camera monitoring and field investigation results, the Siberian roe deer browsed lightly (10%–20%) on the saplings, foraging on ports 1–3 cm from the newborn lateral branch of the current year with inconspicuous traces and small foraging ports. The forage intensity of wapiti was high (70%–100%), and most of the branches and leaves and the foraging ports were not neat (Figure 8).

### 3.4 Effects of Foraging by Two Species of Deer on Japanese Yew Saplings

**FIGURE 9:**
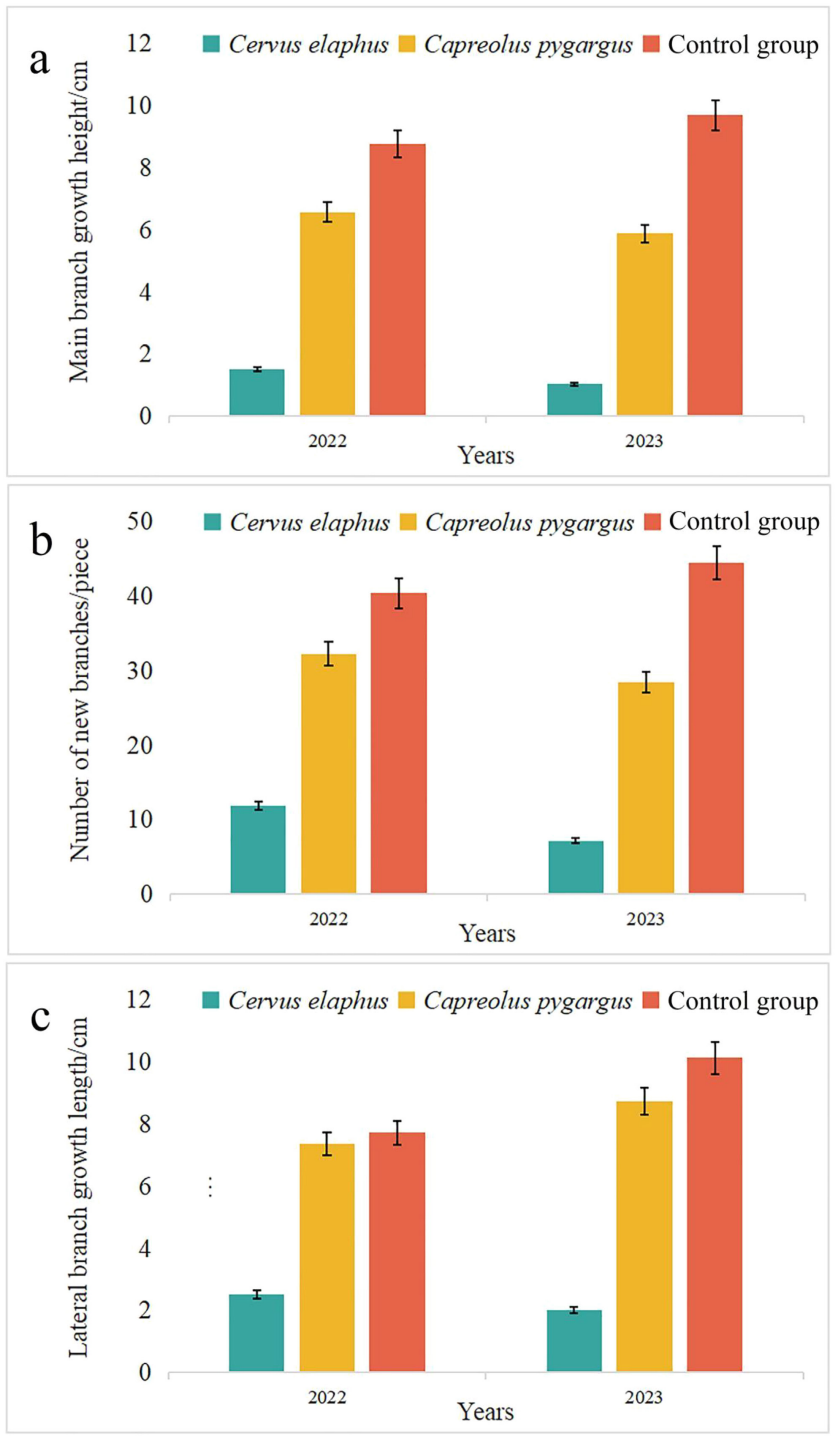
Effects of foraging on saplings of Japanese yew.

Significant differences (*p* < 0.05) were observed in the average growth height of the main branches, the number of new branches, and the length of lateral branches of the saplings after foraging by the two ungulates. All the above variables (average growth height of the main branches, number of new branches, and length of lateral branches) decreased after foraging. There were significant differences between the wapiti and control group (*p* < 0.05), while the differences were not significant between the Siberian roe deer and control group (*p* > 0.05) (Figure 9). These results indicate that the influence of the wapiti foraging on Japanese yew saplings is much greater than that of the Siberian roe deer.

## 4 Discussion

### 4.1 Temporal Strategies for Foraging on the Japanese Yew in Two Deer Species

In the present study, infrared camera detection revealed differences in the temporal strategies of two ungulates foraging on Japanese yew saplings. The daily feeding rhythm of wapiti has two peaks, and that of Siberian roe deer has three peaks, and the peak size of the two deer species is significantly different. According to the results of this study, the daily feeding rhythm of wapiti has obvious regularity, which may be due to temperature extremes in winter; wapiti's peak daily activity usually occurs during the warmer period before sunset. The feeding rhythm of Siberian roe deer is relatively average; it forages almost all day long. There was a large nocturnal difference between the two ungulates, which may be a competitive strategy.

The seasonal variation in foraging activities of wapiti foraging in Japanese yew was significantly greater than that of Siberian roe deer, suggesting that the two deer species may have achieved a certain degree of coexistence in foraging activities by reducing the time overlap with their competitors (Monterroso, Alves, and Ferreras 2014; Romero‐Muñoz et al. 2010; Rheingantz et al. 2016). The foraging frequency of wapiti and Siberian roe deer during the warm season was significantly lower than that during the cold season. The possible reasons are as follows: (1) The temperature is suitable and food resources are abundant in the summer. They can replenish nutrition with other types of green plants, thereby reducing their foraging in Japanese yew. (2) In summer, the forest vegetation is dense, and the Japanese yew is relatively concealed, which is not easy to be found by the two species of deer, thus reducing the risk of being eaten to a certain extent. (3) Japanese yew is one of the few green plants grown in winter that has higher energy, protein, and carbohydrate contents than other plants. Wapiti prefers to eat foods high in protein, carbohydrates, and energy in winter (Huang 2015). Therefore, the selectivity of wapiti to Japanese yew in cold season was greater than that in the warm season.

### 4.2 Foraging Spatial Strategies of the Two Ungulates

Different habitat selection and utilization strategies are key factors that promote the distribution of sympatric animals (Liu et al. 2020; Xia et al. 2019). The prediction results demonstrated that the wapiti foraged on Japanese yew saplings at an altitude of 700 m, with slopes of 20°–30°, aspects to the north, and far away from the road (2000 m) and the water source (3500 m) in the reserve. The Siberian roe deer preferred an altitude of 650 m, with slopes of 15°–20°, and the other factors were similar to the wapiti. In general, there are some similarities between wapiti and Siberian roe deer in the spatial strategies of foraging Japanese yew.

The population size and distribution of ungulates are inextricably linked to plant resources (Wang, Meng, et al. 2019), and they often face trade‐offs between high‐quality food and food abundance in winter, which is related to the spatial distribution of food resources (Van Beest et al. 2010). The prediction results of this study showed that vegetation type was the most important factor affecting the habitat suitability of the two species of deer foraging for Japanese yew and was one of the main sources of competition. Coniferous vegetation is critical to ungulate feeding habitat, and proximity to these forests increases the likelihood of their presence. Both wapiti and Siberian roe deer in the reserve had a greater preference for coniferous forest and broadleaf forest than for pure coniferous forest and pure broadleaf forest, mainly due to differences in the quantity and quality of food provided by different forest types. The species and quantity of plants under the coniferous mixed forest are relatively rich and diverse (including Japanese yew), the pure coniferous forest is dominated by artificial red pine or larch, and the pure broad‐leaved forest is dominated by Mongolian oak. The species and composition of understory shrubs are relatively simple, and the food resources of the two species of deer are relatively low. The response curves of the environmental variables demonstrated that suitable foraging areas are weakly avoided roads and farmland and are insensitive to human activities. This is because the Japanese yew is distributed far from roads and residential areas, and the frequency of human activity is low.

Judging from the results, the reason that the suitable foraging area of Siberian roe deer is much larger than that of wapiti may be related to the large population size, strong reproductive ability, and wide foraging range of Siberian roe deer. According to the analysis of habitat overlap between wapiti and Siberian roe deer, about 65% of the habitats suitable for eating Japanese yew in winter are also suitable for eating areas of Siberian roe deer, which is mainly reflected in the utilization of the mixed forests in the protected area, indicating that the habitats suitable for eating Japanese yew in winter between wapiti and Siberian roe deer has certain similarities. A study on the winter habitat selection of roe deer and muntjac in a commercial forest in Thetford, England, shows that the Pianka's index is 0.40 and 0.55, respectively, at different forest types and different forest growth stages. Pianka's index is higher, and in the event of food shortages, there is a potential competition between the two species for resource use (Hemami, Watkinson, and Dolman 2004). The wapiti and Siberian roe deer have similar ecological niches. As a kind of small and medium‐sized deer, therefore, the Siberian roe deer is vulnerable to the competitive substitution of wapiti in resource utilization (Latham 1999).

### 4.3 Foraging Characteristics of Two Ungulates and the Effects on Saplings

In the long term, vegetation system dynamics may depend on seedling replenishment. A study of alpine plant communities found that trampling and foraging by large and small herbivores negatively affected plant communities (Feng 2022). During the study, it was found that the foraging behavior of animals was one of the reasons for the reduction of the number of individuals in the regeneration layer of Japanese yew population (Diao et al. 2020; Yang 2018), which would be detrimental to the development and recovery of individual yew populations in Japanese yew (Zhou, Liu, and Yuan 2004). In the study on the diet of the wapiti and Siberian roe deer, it is found that Japanese yew is one of the main foods of wapiti in winter, and *Acer tegmentosum* also accounts for a large proportion. Siberian roe deer also eat a certain amount of Japanese yew in winter, but the proportion is small; the main food is *Acer tegmentosum* and *Populus cathayana* (Huang 2015; Zhong 2020; Zhu et al. 2019). This suggests that the effect of Japanese yew on wapiti in winter is greater than that of Siberian roe deer.

The extent to which wapiti forage for Japanese yew belongs to heavy foraging. In addition, the wapiti looks for fallen trees, forage for several branches and leaves, and forage on as many mature tree branches as possible, which were found to be 3 m from the ground. Foraging of roe deer (*Capreolus capreolus*) has impacted the growth of saplings, which accumulate over time, and three consecutive years of foraging reduces growth height by 1 year (Bergquist, Bergström, and Zakharenka 2003; Olofsson et al. 2004). This current study received similar results. The average growth height of the main branches, number of new branches, and length of the lateral branches of the saplings decreased after foraging by both ungulates. The damage degree of the wapiti was much greater than that of the Siberian roe deer. Saplings that were heavily foraged on by wapiti branched out from the root. This may be due to the strong selectivity of wapiti in winter, making it difficult for saplings to grow beyond their foraging height. It may be considered that foraging by large herbivores affected the growth and survival rate of the saplings, causing damage. Considering their special biological characteristics, such as slow growth, poor stress resistance, and low survival rates (Yang 2018), foraging by ungulates seems to aggravate this phenomenon, which is not conducive to normal growth and may affect the natural regeneration of populations. In the short term, there are obvious differences between the two ungulates in foraging and the impact on saplings, which have a certain negative impact on their growth; however, the impact on succession and renewal needs further study.

## 5 Conclusion

The wapiti and Siberian roe deer reduce or avoid direct competition for their resources through different foraging strategies in the Muling National Nature Reserve to ensure their nutritional requirements in the harsh winter environment. Temporally, they also preferred to forage for Japanese yew saplings during the cool season, with a moderate degree of overlap. Spatially, suitable foraging habitats of the Siberian roe deer were approximately twice those of the wapiti, and their overlap was less in location, direction, and distance from mature trees. Behaviorally, they have different foraging intensities, and the influence of wapiti on the growth of saplings is much greater than that of the Siberian roe deer. Therefore, it is recommended that local conservation management departments take necessary measures, such as setting up purse Seine nets and artificial cultivation of other plants (*Euonymus*, *Acer tegmentosum*) for deer foraging options in winter, so as to protect the scattered wild Japanese yew saplings. It is further recommended to provide favorable conditions for the succession and renewal of the forests of Japanese yew without affecting the habitat activities of the wapiti and the normal recovery of the trees to maintain the stability of the forest ecosystems and biodiversity of the region.

## Author Contributions

**Xianzhe Wang:** conceptualization (equal), data curation (equal), formal analysis (equal), funding acquisition (equal), investigation (equal), methodology (equal), project administration (equal), resources (equal), software (equal), supervision (equal), validation (equal), visualization (equal), writing – original draft (equal), writing – review and editing (equal). **Jianan Feng:** conceptualization (equal), data curation (equal), formal analysis (equal), funding acquisition (equal), investigation (equal), methodology (equal), project administration (equal), resources (equal), software (equal), supervision (equal), validation (equal), visualization (equal), writing – original draft (equal), writing – review and editing (equal). **Yang Hong:** writing – original draft (supporting), writing – review and editing (supporting). **Hairong Du:** writing – original draft (supporting), writing – review and editing (supporting). **Minghai Zhang:** conceptualization (supporting), data curation (supporting), formal analysis (supporting), funding acquisition (supporting), investigation (supporting), methodology (supporting), project administration (supporting), resources (supporting), software (supporting), supervision (supporting), validation (supporting), visualization (supporting), writing – original draft (supporting), writing – review and editing (supporting).

## Conflicts of Interest

The authors declare no conflicts of interest.

## Supporting information


Data S1.

## Data Availability

The relevant data of this paper can be found in the Supporting Information.
